# Measles outbreaks in Nepal: sustaining rapid response through equity-focused immunization and surveillance

**DOI:** 10.1097/MS9.0000000000005189

**Published:** 2026-06-05

**Authors:** Alisha G C, Binod G C

**Affiliations:** a Nepalgunj Medial College, Banke, Nepal; b Department of Infection and Immunology, Kathmandu Research Institute for Biological Sciences (KRIBS), Lalitpur, Nepal; c Department of Biomedical Sciences, Cedars-Sinai Medical Center, Los Angeles, CA, USA

**Keywords:** disease surveillance, health equity, immunization, measles outbreak, Nepal

## Abstract

Recent measles outbreaks in Nepal’s Sarlahi and Baglung districts show persistent subnational immunity gaps despite national progress toward measles elimination. This letter supports Nepal’s rapid and coordinated outbreak response, particularly in Sarlahi, where early clinical suspicion, laboratory confirmation within 72 hours, household investigation, active case search, vitamin A supplementation, and outbreak response immunization were promptly implemented. These actions demonstrate the value of functional surveillance, laboratory capacity, and multisectoral coordination. However, the outbreaks also reveal that administrative declarations of high or full immunization coverage may mask susceptibility among marginalized and mobile populations. Cases clustered among under-immunized children in poor Muslim, Dalit, and migrant-linked communities, highlighting measles as a sensitive tracer of inequity in immunization systems. Sustained elimination will require ward-level microplanning, active identification of zero-dose and under-vaccinated children, catch-up vaccination for older cohorts, strengthened risk communication, and engagement of trusted community and religious leaders. Nepal’s recent response provides an encouraging model, but long-term success depends on converting rapid outbreak control into preventive, equity-focused routine immunization.

*Dear Editor*,

We read with great interest the recent reports describing Nepal’s measles outbreaks in Sarlahi and Baglung districts and the rapid public-health actions undertaken to contain transmission. These events are a timely reminder that measles elimination depends not only on high national immunization coverage but also on the ability to identify and close localized immunity gaps among marginalized, mobile, and underserved communities.

The response in Malangwa Municipality, Sarlahi District, deserves recognition. A suspected measles case was reported on 6 January 2026, a blood sample was collected and transported to the National Public Health Laboratory the same day, and laboratory confirmation was received within 72 hours. After further suspected cases were reported on 20 January, field investigation, vitamin A supplementation, laboratory confirmation, outbreak declaration, and outbreak response immunization were rapidly activated. The subsequent campaign targeted children aged 6 months to under 15 years in six high-priority wards, with independent monitoring and follow-up of missed children. This sequence demonstrates the value of functioning case-based surveillance, laboratory capacity, rapid response teams, and local government coordination^[^1^]^.

At the same time, the outbreaks expose a central vulnerability in measles control: aggregate immunization indicators can obscure pockets of susceptibility. In Sarlahi, 34 cases were identified, mostly among unvaccinated or partially vaccinated children, despite the broader provincial claim of full immunization. The Kathmandu Post reported that Madhesh Province had been declared fully immunized in July 2024, yet the Sarlahi outbreak occurred less than 18 months later, with officials acknowledging long-standing immunization gaps in poor and marginalized communities^[^2^]^. This distinction is critical. A province, district, or municipality may appear protected based on administrative data, while specific settlements remain susceptible enough to sustain measles transmission.

The Baglung outbreak further supports this interpretation. Reports indicate that the index case had recently returned from India, and by 31 March 2026, 136 measles cases had been reported in the district, with no deaths recorded at that time. Both Sarlahi and Baglung outbreaks affected socially marginalized communities, including a poor Muslim community in Sarlahi and a Dalit community in Baglung ^[^3^]^. These patterns reinforce the need to treat measles as a tracer condition for inequity in immunization systems. Because measles is among the most contagious human infections, it often reveals the precise locations where routine services, risk communication, migrant follow-up, and community trust have not been sufficient.

The evidence from Nepal’s recent literature is consistent with these observations. A 2026 mixed-methods study of measles-rubella surveillance in Bagmati Province found that the 95% second-dose measles-rubella coverage target was not achieved in most selected municipalities. The study also identified barriers, including limited awareness of vaccine-preventable disease surveillance, poor understanding of the measles elimination target, lack of clarity on standard case definitions, delayed reporting, and limited use of surveillance data^[^4^]^. These findings suggest that elimination requires more than periodic campaigns; it requires routine, ward-level surveillance intelligence that can identify missed children, under-immunized cohorts, and service-delivery bottlenecks before outbreaks occur.

Nepal’s recent outbreaks also fit a broader post-pandemic pattern in which missed routine immunizations, reduced access to health services, and misinformation have slowed progress toward measles elimination. Previous commentary on Nepal’s measles resurgence after COVID-19 emphasized the role of incomplete vaccination, vaccine hesitancy, and access barriers in sustaining outbreaks among vulnerable communities^[^5^]^. Globally, measles continues to function as a sensitive indicator of immunization-system weaknesses; despite substantial mortality reductions since 2000, recent increases in cases and outbreaks show that elimination requires sustained two-dose coverage, strong surveillance, and targeted closure of immunity gaps^[^6^]^.

Nepal’s previous successes show that this goal is achievable. The country has built substantial immunization infrastructure, including laboratory-supported surveillance, supplementary immunization activities, and community-based outreach. Nepal was verified as having eliminated rubella in 2025, reflecting strong political commitment, health-worker engagement, and partner-supported surveillance systems^[^7^]^. Similarly, Nepal’s 2020 measles outbreak response immunization campaign, implemented during COVID-19 transmission, vaccinated more than 32 000 people with reported 97% coverage and was associated with a 98% decrease in measles incidence after the response^[^8^]^. These examples indicate that Nepal has the operational foundation needed for measles elimination if the same intensity is sustained in routine immunization and catch-up activities.

Community engagement should remain central. The Sarlahi response included engagement with religious leaders, teachers, and Female Community Health Volunteers to promote measles-rubella vaccination and the reporting of suspected cases^[^1^]^. Experiences from Nepalgunj, Banke District, also show the value of human-centered design approaches for understanding vaccine hesitancy, including fears of side effects, mistrust, cultural beliefs, and gendered decision-making barriers. UNICEF reported that this approach reached 65 households across three wards and supported the door-to-door vaccination of 40 children^[^9^]^. Such approaches are particularly important where biomedical messages alone may not overcome mistrust or access barriers.

We therefore support the rapid and coordinated response demonstrated in Sarlahi, while emphasizing that the next phase must be preventive rather than reactive. Nepal should prioritize ward-level microplanning, active identification of zero-dose and under-immunized children, catch-up vaccination for older children and adolescents, school-based verification of immunization status, and cross-border coordination for mobile populations. The World Health Organization South-East Asia Region’s current strategic plan envisions measles and rubella elimination in the region by 2026 and emphasizes high population immunity, sensitive case-based surveillance, laboratory capacity, rapid outbreak response, and sustained political commitment^[^10^]^.

In conclusion, Nepal’s recent outbreaks should not be interpreted solely as setbacks. They also demonstrate that early detection, laboratory confirmation, community mobilization, and outbreak response immunization can limit transmission when implemented promptly. The challenge now is to convert this rapid-response capacity into a sustained, equity-focused immunization strategy. If Nepal closes subnational immunity gaps, strengthens surveillance at the point of care, and continues to work through trusted community structures, measles elimination remains a realistic and necessary public-health objective.

## Data Availability

Not applicable.

## Research registration unique identifying number (UIN)

Not applicable. This is a Letter to the Editor and does not report original clinical research or human trials requiring registration.
